# Herpes zoster hospitalization as a sentinel signal for undiagnosed dysglycemia: a call for routine glycemic screening in clinical practice

**DOI:** 10.3389/fcdhc.2026.1732223

**Published:** 2026-04-02

**Authors:** Tao Xu, Keying Ji, Xi Pu, Shuangmeng Mou

**Affiliations:** 1 Department of Dermatology, The 945 Hospital of Joint Logistics Support Force of Chinese People’s Liberation Army (PLA), Ya’an, China; 2 Department of Dermatology, Ya’an Polytechnic College Affiliated Hospital, Ya’an, China

**Keywords:** diabetes screening, glycemic control, herpes zoster, hospitalization, undiagnosed dysglycemia

## Abstract

**Background:**

Although diabetes mellitus (DM) is an established risk factor for herpes zoster (HZ), the prevalence of undiagnosed dysglycemia in hospitalized HZ patients, and its association with HZ severity, are not well characterized. This study investigated the prevalence of newly identified diabetes and prediabetes in a cohort of patients hospitalized for HZ, comparing those with disseminated and localized presentations.

**Methods:**

A retrospective cohort analysis of 564 hospitalized patients with HZ from 2020 to 2024 after exclusion was performed. The patients were divided into localized and disseminated HZ groups based on their clinical presentation. Diabetes was diagnosed on the basis of the 2023 American Diabetes Association (ADA) criteria. Multivariable logistic regression was used to identify independent risk factors for diabetes.

**Results:**

Among 564 hospitalized HZ patients, 45 were diagnosed with disseminated HZ. Disseminated HZ was associated with a significantly higher prevalence of newly identified diabetes (22.2% [10/45] vs. 11.9% [62/519], P = 0.048) and prediabetes (33.3% [15/45] vs. 13.1% [68/519], P<0.001), as well as poorer chronic glycemic control (median HbA1c 8.90% vs. 7.50%, P = 0.021).Multivariable logistic regression model confirmed disseminated HZ as an independent predictor of diabetes (adjusted OR = 2.06, 95% CI 1.15–3.69), alongside age >60 years and BMI >25 kg/m². Elevated LDL-C and triglycerides were also significant independent risk factors.

**Conclusion:**

Our study identifies disseminated HZ as a key sentinel for previously unrecognized dysglycemia. Routine glycemic screening should be integrated into admission protocols for all hospitalized HZ patients, particularly for severe cases.

## Introduction

Herpes zoster (HZ), caused by the reactivation of varicella-zoster virus (VZV), affects approximately 1 million people annually in the United States alone, with complications such as postherpetic neuralgia (PHN) causing debilitating pain in 10-15% of cases and significantly reducing quality of life (1, 2). The risk of HZ increases with age and immunocompromised status, but diabetes mellitus (DM) has emerged as a critical modifier. Epidemiological studies have consistently demonstrated that DM exacerbates HZ susceptibility; for instance, pooled data show that the incidence of HZ in DM patients is 7.22 per 1000 person-years, compared to 4.12 per 1000 person-years in non-DM individuals (3). This increased risk is attributed to multiple pathways, including hyperglycemia-induced impairment of immunity and chronic inflammation, which compromise cell-mediated defenses against VZV reactivation (4, 5).

In China, the public health burden of HZ is substantial and rising, a trend largely attributable to rapid population aging (6). Concurrently, diabetes represents a major health challenge, with a standardized prevalence of approximately 10.9% among Chinese adults (7). Despite the well-established link between dysglycemia and HZ incidence, routine blood glucose screening for hospitalized HZ patients is not standardized in Chinese clinical practice. This gap is critical. Preliminary evidence suggests that only about 36.5% of individuals with diabetes are aware of their diagnosis, implying that a large proportion—nearly two-thirds of cases—remain undiagnosed (8). However, robust data on the prevalence of undiagnosed diabetes and prediabetes specifically among hospitalized HZ patients in China are lacking, particularly for those with severe forms such as disseminated HZ. This scarcity of evidence hinders the optimization of evidence-based inpatient screening protocols.

Current guidelines, including those from the Advisory Committee on Immunization Practices and the American Diabetes Association (ADA), strongly recommend recombinant zoster vaccine for diabetic patients aged ≥50 years, as it is approved for preventing both HZ and PHN (9, 10). However, a critical knowledge gap persists; it remains unknown whether HZ itself, particularly severe forms such as disseminated HZ, could serve as a signal for undiagnosed diabetes, and this possibility is unaddressed in current screening protocols. This bidirectional relationship is mechanistically plausible because VZV reactivation may trigger systemic stress responses that worsen glycemic control (11). Reviews advocate systematic diabetes screening in all patients with HZ due to evidence of poor glycemic control in this population (12).

Although case reports describe incidental hyperglycemia during HZ episodes, large-scale evidence validating HZ as a sentinel for diabetes screening is lacking (13). To address this evidence gap and to investigate the association between HZ severity and undiagnosed dysglycemia, we conducted a 5-year retrospective cohort study of hospitalized HZ patients. We hypothesized that: (1) a substantial proportion of patients hospitalized for HZ would have previously unrecognized dysglycemia, and (2) the prevalence of newly identified diabetes and prediabetes would be significantly higher in those with disseminated HZ compared to those with localized HZ (14).

## Material and methods

### Study design and population

This retrospective cohort study analyzed the electronic health records (EHR) of patients hospitalized with herpes zoster (HZ) at Ya’an Polytechnic College Affiliated Hospital between January 2020 and December 2024. The inclusion criteria were as follows: (1) clinical diagnosis of HZ (typical dermatomal rash with pain) or laboratory-confirmed VZV reactivation (PCR-positive vesicular fluid) and (2) complete glycemic and dermatological documentation. Exclusion criteria were as follows: (1) prior diagnosis of diabetes mellitus; (2) systemic steroid use >10 mg/day prednisone equivalent within 3 months; (3) pregnancy, active malignancy, HIV/AIDS, or immunosuppressive conditions (e.g., organ transplantation, autoimmune disease on biologics); and (4) incomplete demographic or laboratory data. No age restrictions were applied to better reflect the real-world spectrum of hospitalized HZ patients.”

### Data collection and quality control

Data were extracted from the hospital’s structured EHR system, which includes standardized fields for demographics, laboratory results, and coded diagnoses. However, clinical details regarding HZ morphology, certain comorbidities and complications of HZ were manually abstracted from physicians’ narrative notes and discharge summaries. Two trained researchers independently extracted data using a predefined electronic form, with inter-rater reliability assessed by Cohen’s κ (κ=0.92 for HZ morphology classification). The key variables collected included demographic data (age, sex, BMI, smoking status), morphology classified as localized (rash confined to 1–2 dermatomes) or disseminated (rash involving ≥3 dermatomes or visceral organs or > 20 vesicles appearing at sites outside the initial dermatome), comorbidities (history of hypertension or diabetes), and laboratory data (HbA1c, fasting plasma glucose, liver/kidney function, complete blood test, etc.). The data collected by the two researchers were verified and analyzed by a third researcher.

### Diagnosis of newly identified diabetes/prediabetes during hospitalization

Patients were considered to have newly identified diabetes if they met the 2023 ADA diagnostic criteria during this admission (FPG ≥7.0 mmol/L, HbA1c ≥6.5%, or 2-h OGTT ≥11.1 mmol/L) and had no documented history of diabetes prior to admission (15). The term “newly identified” specifically refers to the first-time diagnosis within the healthcare system during this hospitalization. To minimize misclassification due to acute illness-related stress hyperglycemia, the following steps were taken: (1) FPG was measured immediately upon admission and confirmed with a repeat measurement after 48 hours; (2) For patients with elevated FPG but HbA1c <6.5%, an OGTT was performed after acute symptoms subsided (before discharge) to confirm the diagnosis; (3) All diagnostic tests were reviewed and confirmed by an endocrinologist. Patients were considered to have newly identified prediabetes if they met the 2023 ADA criteria during this admission (impaired fasting glucose [IFG: FPG 6.1–6.9 mmol/L] or impaired glucose tolerance [IGT: 2-hour OGTT 7.8–11.0 mmol/L]) and had no prior history of dysglycemia.

### Statistical analysis

Statistical analyses were performed with SPSS Statistics for Windows version 26.0 (IBM). To evaluate the normality of the data, both the Shapiro–Wilk test and histograms were used. Continuous variables are expressed as medians or interquartile ranges, and categorical variables are expressed as numbers and percentages. For a two-group comparison, continuous variables were compared using the t-test or Mann-Whitney U test, while categorical variables were compared using the chi-square test or Fisher-Freeman-Halton exact test, where appropriate. Multivariable logistic regression was used to analyze potential risk factors for newly identified diabetes in patients with HZ. Covariates for the initial multivariable model were selected based on clinical relevance, all predictor variables were collected at admission or within the first 24 hours, representing baseline characteristics. A two-sided P value < 0.05 was considered statistically significant for all analyses.

## Results

**Figure 1:**
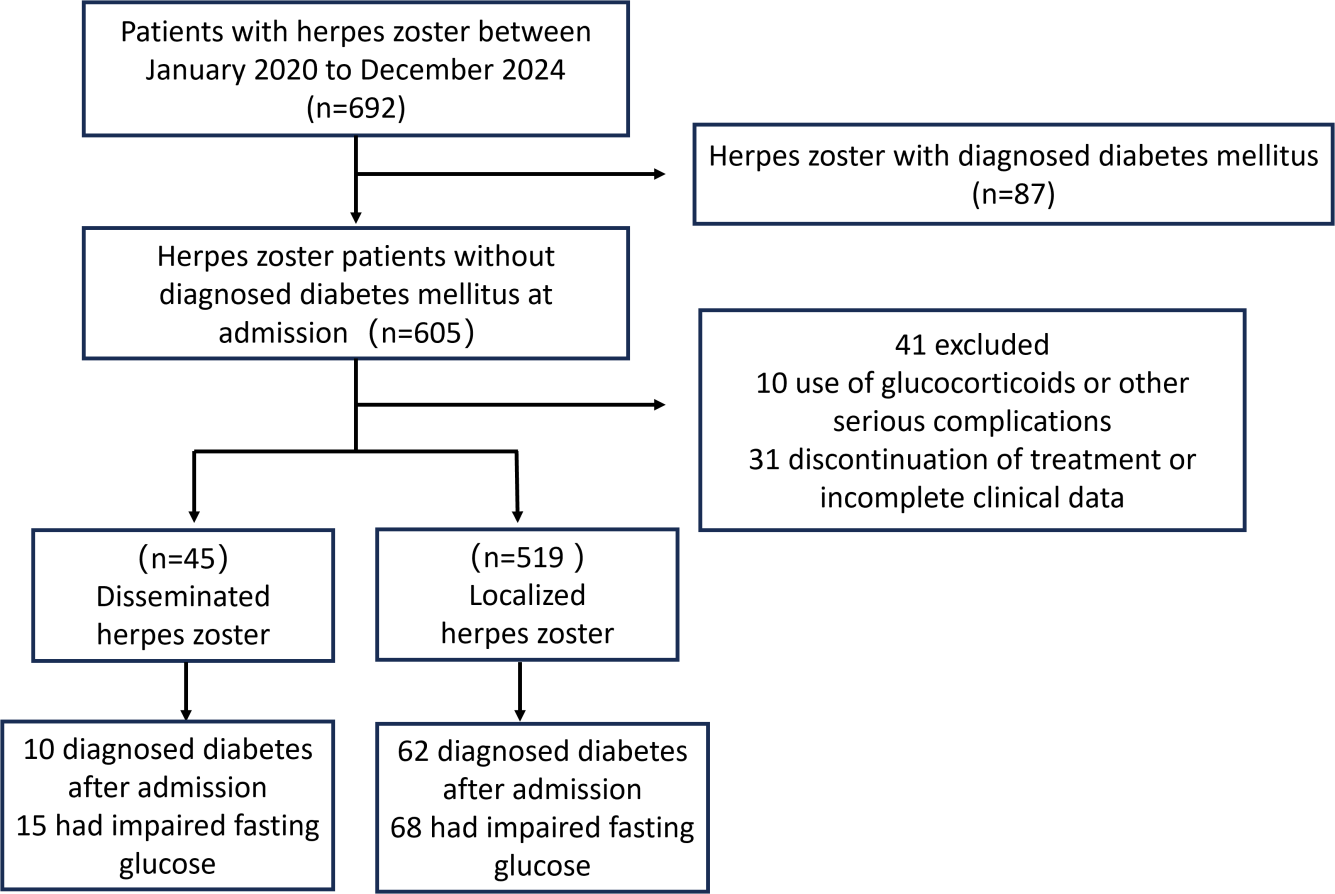
A comprehensive flow chart of patients through this retrospective study.

A total of 692 patients hospitalized for herpes zoster between January 2020 and December 2024 were evaluated. 87 were excluded for pre-existing diabetes, 10 for glucocorticoid use/comorbidities, and 31 for incomplete data, leaving 564 patients who were eligible for the study (**Figure 1**). Of the total patients, 45 (8.0%) had disseminated HZ, and 519 (92.0%) had localized HZ. The age of the study population ranged from 30 to 87 years.

Patients with disseminated HZ were significantly older (mean age 63.5 ± 8.7 years vs. 59.8 ± 9.2 years, P = 0.009) and had a higher mean BMI (25.8 ± 2.9 kg/m² vs. 24.3 ± 3.1 kg/m², P = 0.002) than the localized HZ group. The distribution of lesion severity differed markedly between the groups (P<0.001): disseminated HZ patients had substantially higher rates of hemorrhagic vesicles (11.1% vs. 5.0%) and necrosis/visceral involvement (8.9% vs. 0.1%) as the most serious lesion type. No significant differences were found between the groups in sex distribution (P = 0.624), smoking status (P = 0.389), prevalence of comorbidities (P = 0.158), frequency of HZ complications (P = 0.350), and time from symptom onset to hospital admission (5.0 (4.0~7.0) vs. 4.0 (3.0~6.0) days, P = 0.139) (**Table 1**).

**Table 1:** Baseline characteristics of the study population.

Variable	Localized HZ (n=519)	Disseminated HZ (n=45)	Test statistic value	P-value
^1^Age (years)	59.8 ± 9.2	63.5 ± 8.7	-2.62	0.009
^2^Gender			0.24	0.624
Male	268 (51.6%)	25 (55.6%)		
Female	251 (48.4%)	20 (44.4%)		
^1^BMI(Kg/m^2^)	24.3 ± 3.1	25.8 ± 2.9	-3.18	0.002
^2^Smoker			0.86	0.389
Yes	195 (37.6%)	14 (31.1%)		
No	324 (62.4%)	31 (68.9%)		
^3^The most serious type of lesions			16.24	<0.001
Erythema and blisters	395 (76.1%)	26 (57.8%)		
Purulent vesicles	93 (17.9%)	10 (22.2%)		
Hemorrhagic vesicles	26 (5.0%)	5 (11.1%)		
Necrosis/visceral organs involvement	5 (1.0%)	4 (8.9%)		
^2^Comorbidities			1.99	0.158
Yes	243 (46.8%)	26 (57.8%)		
No	276 (53.2%)	19 (42.2%)		
^2^Complications of HZ			0.87	0.350
Yes	172 (33.1%)	18 (40.0%)		
No	347 (66.9%)	27 (60.0%)		
^4^Time from HZ onset to hospital admission (days)	5.0 (4.0~7.0)	4.0 (3.0~6.0)	-1.48	0.139

HZ, Herpes Zoster; BMI, Body mass index; Complications of HZ, Peripheral Nerve Palsies, Keratitis, Uveitis, Bacterial Superinfection, Urinary retention, etc. ^1^t-test, ^2^Chi-square test, ^3^Fisher-Freeman-Halton exact test, ^4^Mann-Whitney U test.

**Figure 2:**
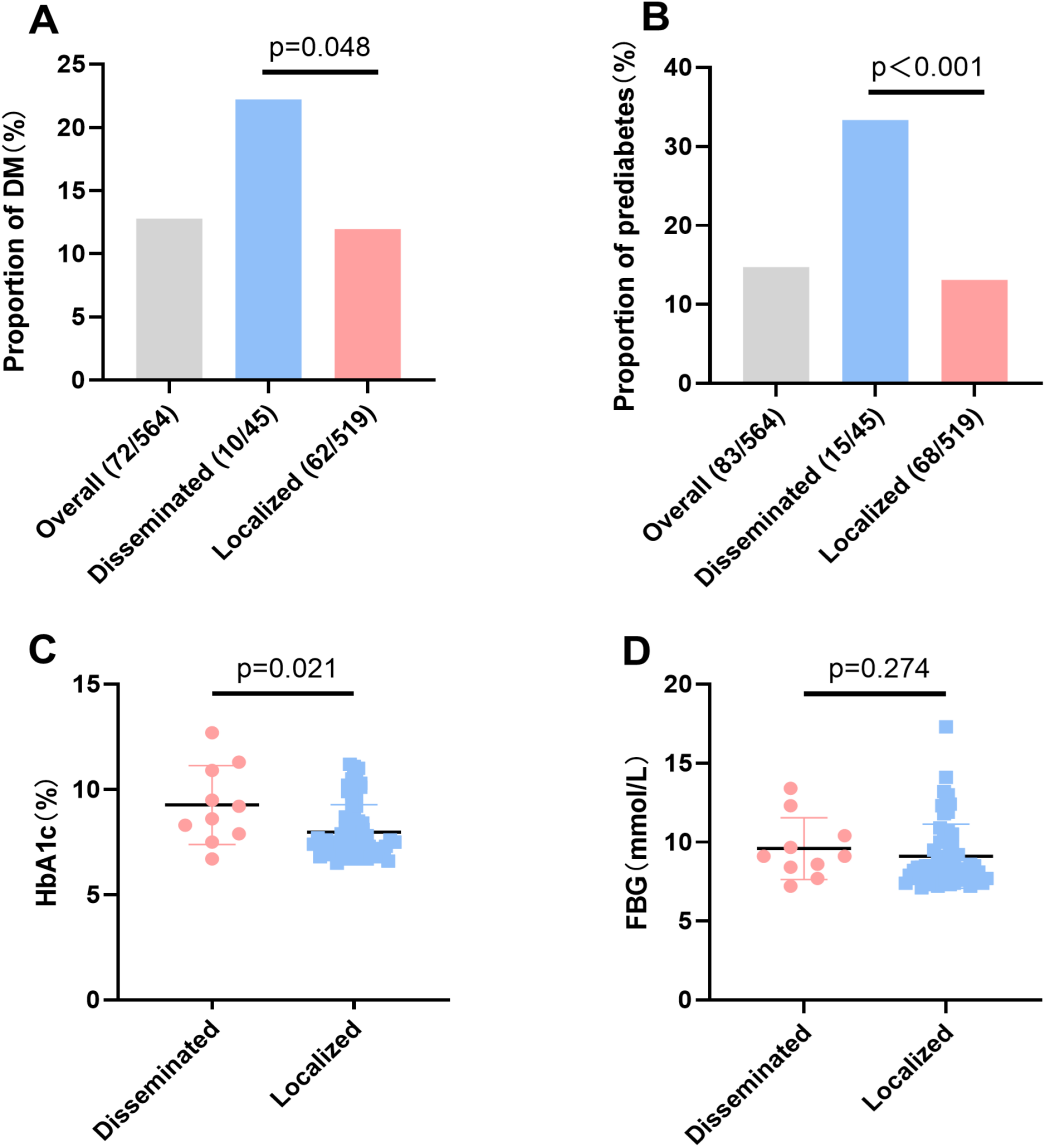
Distribution and glycemic profiles in HZ patients. **(A)** Comparison of the proportions of newly identified diabetes among disseminated and localized HZ patients. **(B)** Comparison of the proportions of prediabetes among disseminated and localized HZ patients. **(C)** Comparison of HbA1c Levels in patients with newly identified diabetes. **(D)** Comparison of fasting blood glucose in patients with newly identified diabetes. HZ, Herpes Zoster; DM, Diabetes Mellitus; HbA1c, Glycated Hemoglobin; FBG, Fasting Blood Glucose. **(A, B)** Chi-square test, **(C, D)** Mann-Whitney U test.

Newly identified diabetes was identified in 10/45 (22.2%) disseminated HZ patients and in 62/519 (11.9%) localized HZ patients (p=0.048). A higher rate of prediabetes was observed in patients with disseminated HZ (33.3%, 15/45) than in those with localized HZ (13.1%, 68/519) (P < 0.001) (**Figures 2A, B**).

Among patients with newly identified diabetes, those presenting with disseminated HZ showed significantly poorer glycemic control than those with localized HZ. The median HbA1c level was significantly higher in disseminated HZ patients (8.90%, IQR 7.80–11.00) compared with localized HZ patients (7.50%, IQR 7.00–8.53) (P = 0.021, **Figure 2C**). Fasting blood glucose (FBG) levels, though numerically higher in disseminated HZ (9.10 mmol/L vs. 8.35 mmol/L, IQR 8.23–10.88 vs 7.70–10.05), did not reach statistical significance (P = 0.274, **Figure 2D**).

Multivariable logistic regression analysis confirmed disseminated HZ as an independent risk factor for newly identified diabetes (OR = 2.06, 95% CI 1.15–3.69, P = 0.014). Other independent predictors identified in the final model included age >60 years (OR = 2.00, 95% CI 1.39–2.87, P<0.001), BMI >25 kg/m² (OR = 1.79, 95% CI 1.27–2.51, P = 0.001), elevated low-density lipoprotein cholesterol (LDL-C >3.4 mmol/L; OR = 1.75, 95% CI 1.28–2.40, P<0.001), and elevated triglycerides (TG >1.7 mmol/L; OR = 1.47, 95% CI 1.10–1.95, P = 0.009). Variables such as sex, smoking status, hypertension, other comorbidities, HZ complications, elevated creatinine, and elevated transaminase levels did not show statistically significant independent associations in this model (all P>0.05) (**Table 2**).

**Table 2:** Multivariable logistic regression analysis of risk factors for newly identified diabetes in HZ patients.

Variables	β Coefficient	OR	95% CI	P-value
Gender				0.477
Female		1.000	reference	
male	0.108	1.114	0.828-1.499	
Age (years)				<0.001
<60		1.000	reference	
≥60	0.692	1.998	1.391-2.870	
BMI (kg/m²)				0.001
<25		1.000	reference	
≥25	0.580	1.786	1.273-2.506	
Smoker				0.617
No		1.000	reference	
Yes	0.070	1.073	0.815-1.412	
Hypertension				0.755
No		1.000	reference	
Yes	0.048	1.049	0.776-1.419	
Disseminated HZ				0.014
No		1.000	reference	
Yes	0.725	2.064	1.154-3.691	
Other Comorbidities				0.520
No		1.000	reference	
Yes	0.092	1.096	0.828-1.452	
Complications of HZ				0.678
No		1.000	reference	
Yes	-0.061	0.941	0.705-1.256	
Elevated Creatinine				0.510
No		1.000	reference	
Yes	0.128	1.137	0.777-1.664	
Elevated Transaminase				0.465
No		1.000	reference	
Yes	0.119	1.126	0.818-1.550	
Elevated LDL-C				<0.001
No		1.000	reference	
Yes	0.560	1.751	1.280-2.396	
Elevated TG				0.009
No		1.000	reference	
Yes	0.382	1.465	1.100-1.951	

BMI, body mass index; CI, confidence interval; HZ, herpes zoster; LDL-C, low-density lipoprotein cholesterol; OR, odds ratio; TG, triglycerides.

Definitions: “Other comorbidities” refers to major chronic conditions excluding hypertension, such as coronary artery disease, chronic kidney disease, or cerebrovascular disease. Elevated laboratory parameters were defined according to common clinical reference thresholds in China: Elevated Creatinine (>97 μmol/L for males, >73 μmol/L for females). Elevated Transaminases (alanine aminotransferase [ALT] >40 U/L or aspartate aminotransferase [AST] >40 U/L). Elevated LDL-C (>3.4 mmol/L), and Elevated TG (>1.7 mmol/L).

## Discussion

Our study underscores a critical clinical observation: a substantial proportion of hospitalized patients with herpes zoster (HZ) harbor previously undiagnosed dysglycemia, with the prevalence being notably higher in cases of disseminated HZ. This finding aligns with and extends prior epidemiological evidence. Numerous studies have established diabetes mellitus as a significant risk factor for HZ, reporting an increased incidence among diabetic individuals (16–19). Furthermore, evidence suggests that close to 30% of HZ cases may occur in individuals with undetected hyperglycemia, indicating that underlying dysglycemia contributes to HZ susceptibility even before a formal diabetes diagnosis is established (20). Collectively, our findings reinforce the potential of HZ hospitalization as a sentinel opportunity to identify and diagnose underlying diabetes that might otherwise remain unrecognized.

Our multivariable analysis identified several independent predictors for newly identified diabetes. In line with established epidemiology, older age, higher BMI, and elevated lipid levels were significantly associated with increased risk. Crucially, disseminated HZ remained a strong independent predictor after adjusting for these metabolic and demographic factors. In contrast, smoking status was not a significant independent predictor in this model, which may be attributable to the relatively low prevalence of active smokers in our cohort or may suggest that in the acute inpatient setting, the influence of pronounced gluco-metabolic disturbances predominates.

Among these identified risk factors, the association of dyslipidemia with newly identified diabetes in our cohort warrants further consideration. While dyslipidemia is a well-recognized component of the metabolic syndrome and often precedes or coexists with glucose metabolism disorders (21), its role in the context of acute HZ is less understood. It is plausible that lipid abnormalities contribute to a pro-inflammatory state that exacerbates HZ severity, thereby increasing the likelihood of unmasking underlying dysglycemia (22). Alternatively, these lipid disturbances may simply reflect the broader metabolic dysregulation in individuals predisposed to both conditions. Future studies are needed to elucidate whether lipid-lowering therapies could modify the risk of HZ complications or associated glycemic decompensation.

In disseminated HZ, intensified viral dissemination likely exacerbates immunometabolic stress, revealing a bidirectional relationship between dysglycemia and disease severity. On one hand, hyperglycemia-induced impairment of cell-mediated immunity facilitates VZV reactivation and propagation, predisposing patients to more severe clinical presentations (23). On the other hand, VZV infection itself may contribute to glycemic dysregulation through immune-mediated mechanisms. Viral replication triggers a robust innate and adaptive immune response, characterized by the release of type I interferons and pro-inflammatory cytokines such as TNF-α and IL-6. These immune mediators have been shown to disrupt insulin signaling in peripheral tissues, leading to decreased insulin sensitivity and increased hepatic glucose production (24). Experimental evidence further supports this pathway: viral-induced inflammation can induce skeletal muscle insulin resistance and, in susceptible models, precipitate glucose intolerance (25). This immunometabolic interplay creates a self-perpetuating cycle that helps explain why patients with disseminated HZ exhibit both a higher prevalence of newly identified dysglycemia and poorer glycemic control compared to those with localized disease.

The clinical significance of this self-perpetuating cycle is underscored by evidence linking poor glycemic control to worse outcomes. Research indicates that elevated HbA1c levels correlate strongly with protracted PHN severity and extended viral shedding in HZ patients, reflecting how hyperglycemia may compromise endothelial function and skin immunity, creating a vicious cycle of metabolic and viral dysregulation (26, 27). One study demonstrated that HZ patients with poor glycemic control (HbA1c >9.0%) experienced more intense and prolonged pain than those with good control, reinforcing our hypothesis that disseminated HZ represents a critical window in which glycemic instability amplifies disease severity (28).

Our findings indicate that not only is newly diagnosed diabetes more frequent in patients with disseminated HZ but the prevalence of prediabetes is also markedly elevated in this subgroup. This is consistent with previous investigations linking abnormal glycemic status (both high and low HbA1c levels) to heightened HZ susceptibility (29). For instance, studies evaluating glycemic status found that individuals with HbA1c >10.3% or <5.0% had significantly increased HZ risk compared to those with good HbA1c control (HbA1c 5.0-6.7%), suggesting that dysglycemia, including prediabetes, serves as a key immunometabolic risk factor (30).

Clinically, these insights advocate for proactive screening and prevention strategies. The high rate of occult diabetes and prediabetes in HZ, especially in disseminated cases, supports the integration of routine DM screening into standard HZ management protocols. Current guidelines already advocate multifactorial disease management in aging cohorts, and our data strengthen the case for targeted glycemic assessments in all HZ patients to mitigate complications (31). Simultaneously, vaccination has emerged as a vital intervention. Recombinant zoster vaccines have demonstrated >90% efficacy in preventing HZ and PHN across diverse populations, including those with DM (32, 33). This aligns with the recommendations that diabetic patients prioritize vaccination to counteract their inherent susceptibility, potentially averting severe outcomes, such as dissemination and prolonged neuralgia (34).

Our study has several limitations. First, its single-center, retrospective design may introduce selection bias and limit generalizability. Second, due to the cross-sectional nature, we can identify associations but cannot establish causality or analyze time-to-event. Third, the diabetes-related data collected were restricted to essential diagnostic parameters and lacked a detailed metabolic profile. Future large-scale, prospective cohorts with comprehensive metabolic profiling and longitudinal follow-up are needed to validate our findings and better define the temporal relationship between HZ severity and dysglycemia.

## Conclusion

Disseminated herpes zoster serves as a significant sentinel signal for newly identified diabetes and prediabetes during hospitalization, indicating a strong association with poor chronic glycemic control. Our findings advocate the routine integration of comprehensive glycemic screening into standard admission protocols for all patients hospitalized with HZ, with particular emphasis on those presenting with disseminated disease.

## Data Availability

The raw data supporting the conclusions of this article will be made available by the authors, without undue reservation.
